# Skeleton keys and Trojan horses: a review of therapeutic delivery to the brain

**DOI:** 10.3389/fcell.2025.1674333

**Published:** 2025-10-17

**Authors:** Rachel E. Stoub, Barbara J. Bailus

**Affiliations:** ^1^ Henry E. Riggs School of Applied Life Sciences, Keck Graduate Institute, Claremont, CA, United States; ^2^ Scripps College, Claremont, CA, United States

**Keywords:** blood brain barrier, adeno-associated virus, lipid nanoparticle, receptor mediatedtranscytosis, cell penetrating peptide, focused ultrasound, hematopoietic stem cells

## Abstract

**Background:**

The advances in genetic medicine that have occurred in the last few decades have been tempered by the challenges in delivering those medicines to the desired organs and cell types. Nowhere has this delivery challenge been greater than in the brain, due to the blood brain barrier (BBB), often illustrated as an impenetrable castle wall. As the need for neurological therapies grows, an assortment of Trojan horse and skeleton key strategies have been designed to allow passage of therapeutics through the BBB, These range from designer viral vectors, to cell penetrating peptides that can target cell surface receptors, to genetically modifying hematopoietic stem cells, to lipid nanoparticles that pass through the cell membrane.

**Results:**

This review will examine the precise method that each delivery vehicle uses to enter and transverse the endothelial layer of the to BBB and arrive in the brain parenchyma. The advantages and challenges of each delivery strategy will be discussed, as will the most recent clinical trials using these technologies.

**Conclusion:**

There are several extremely promising delivery vehicles that are able to cross the BBB and deliver genetic therapies to neuronal cells. Several of these delivery vehicles have already been approved for use in patients. As these delivery vehicles become further optimized there is the potential to treat a majority of neurological disease and disorders.

## Introduction

Over three thousand years ago ancient Egyptians viewed the brain as disposable, not warranting any special treatment or embalming during the mummification process. Our understanding of the brain has advanced exponentially in the intervening three thousand years, cumulating in viewing the brain as the most vital and irreplaceable organ in the human body. Evidence of human brain surgery exists as early as the late bronze age with individuals undergoing cranial trephination to relive symptoms associated with either brain injury or disease (Kalisher et al., 2023). Until the sequencing of the human genome in 2003 treatments for different brain disorders were largely focused on symptoms and behavioral interventions, as underlying molecular causes were unknown, with highly invasive surgeries being a routine form of treatment until the mid-1900s (Nurk et al., 2022). The sequencing of the human genome traced many neurological disorders to single genes, making those disorders prime candidates for precise therapeutics (Nurk et al., 2022; International Human Genome Sequencing C, 2004). Currently, the National Institutes of Neurological and Stroke Disorders lists over 400 different neurological diseases and disorders, and there are over 5000 known neurological diseases and disorders afflicting 1 in 3 individuals worldwide, these diseases and disorders are the leading cause of disability adjusted life years and the second leading cause of death (Collaborators, 2019). This represents a substantial unmet need in treating neurological disorders and diseases. Healthcare has advanced tremendously since the 1900s, but one of the great challenges of the 21th century remains the treatment and cure of neurological disorders. Perhaps the greatest challenge in treating brain disorders is also what helps protect the brain from infection, the blood brain barrier (BBB). The blood brain barrier is a complex and extensive network of semipermeable and highly selective blood vessels that are integrated throughout the brain. It enables the passage of essential molecules from the bloodstream into the brain, while barring entrance to a majority of pathogens, and over 98% of potential therapeutics (Teleanu et al., 2022; Wu D. et al., 2023). This challenge has been the focus of substantial research and funding resources over the past several decades including BrainMaps, Allen Brain Atlas, and most recently the BRAIN Initiative, all of these programs have dramatically increased our understanding of the brain structure and function, enabling the creation of better delivery vehicles for transporting therapeutics past the BBB and into neuronal cells.

Following the mapping of the human genome and identifying genes involved in neurological disorders tremendous progress has been made in genetic medicine, allowing for the targeting of specific genes for correction and regulation. The available therapeutic technologies include antisense oligonucleotides (ASOs), gene replacement therapy (cDNA), enzyme replacement therapy (ERT) and gene editing and small molecules. These modalities hold the promise to significantly treat or even cure many neurological disorders, with many being used successfully in patients (Wang JH. et al., 2024; Dornelles et al., 2024; Shchaslyvyi et al., 2023; Torroba et al., 2023; Marchetti et al., 2022; Prakash, 2017). However, for these therapies to reach their full potential they all must overcome the same challenge, efficient and widespread delivery past the BBB. A variety of promising delivery techniques has been validated in animal models, and several have already been used in the clinic or are in late stage pre-clinical development (Teleanu et al., 2022; Abbott, 2025). Amongst these delivery technologies there are four major categories, viral, non-viral, cell carriers and physical disruption of the BBB. Each of these delivery technologies offers benefits and limitations, and each technology uses a different strategy for delivering the desired therapeutic into the brain. This review will examine how the unique structure of the BBB informs the evolving development of therapeutic delivery technologies to circumvent or pass through the BBB and into neuronal, glial and other cell types where the targeted therapies are needed.

## Castle fortifications: structure and cellular composition of the blood brain barrier

**FIGURE 1:**
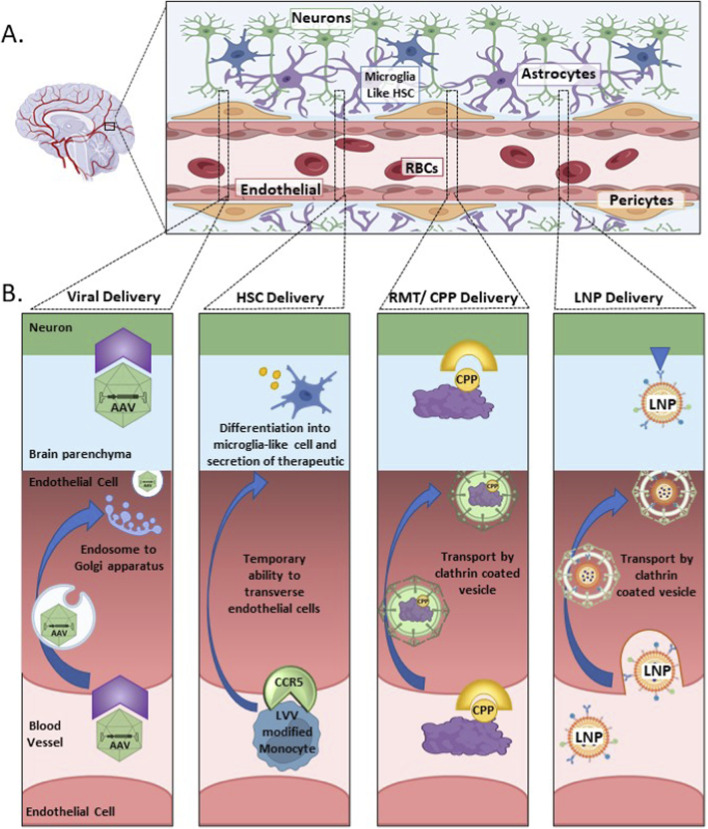
A schematic representing the blood brain barrier (BBB) and associated neurovascular network, with examples of how different delivery vehicles cross the BBB and are able to enter the brain parenchyma. **(A)** The neurovascular network in the brain. **(B)** Illustrating the different strategies used by various delivery vehicles for entering and traversing the endothelial cells of the BBB to reach the brain parenchyma and neuronal cells. Abbreviations used in figure: AAV, adeno-associated virus; CPP, cell penetrating peptide; CCR5, C-C chemokine receptor type 5; HSC, hematopoietic stem cell; LNP, lipid nanoparticle; LVV, lentiviral vector; RBCs, red blood cells.

The BBB is a complex system of vasculature and specialized cells which allow restricted access to the brain protecting the brain from various molecules and pathogens. This system is composed of endothelial cells, astrocytes, and pericytes (Kaya and Ahishali, 2021; Wong et al., 2013). The endothelial cells are connected by tight and adherent junctions, making transport of therapeutics from the blood vessels and into endothelial cells and then neuronal cells difficult. In visualizing this system one can begin at the center with the blood vessels that weave a complex network throughout the entire brain, surrounding the blood vessels are the endothelial cells, then come the pericytes and astrocytes, and further outwards are the neurons and microglia cells (Figure 1) (Wu D. et al., 2023; Gullotta et al., 2023; Alahmari, 2021). This complex network of vessels and surrounding cells that comprise the neurovascular network reaches all regions of the brain, making this an appealing avenue to leverage when delivering therapeutics.

The endothelial cells that line the BBB represent the main point of entrance for therapies that are intended for the brain, they are the “gatekeeper cells”. These specific endothelial cells have several special modifications when compared to peripheral endothelial cells. Unlike peripheral endothelial cells those lining the BBB present with no small transcellular pores, which usually facilitate diffusion exchange of small molecules between cells (Wu D. et al., 2023; Alahmari, 2021; Hennigs et al., 2021). This lack of small transcellular pores contributes to the inability to deliver over 98% of small molecule drugs to the brain by peripheral intravenous injections (Teleanu et al., 2022; Wu D. et al., 2023). Beyond this lack of transcellular pores the endothelial cells are connected to each other by tight and adherens junctions. The use of tight junctions for connecting the endothelial cells is another barrier when trying to deliver therapeutics to the brain. Unlike gap junctions, tight junctions do not facilitate cell to cell connections, but instead limit cell to cell connection to prevent leakage between cells (Kaya and Ahishali, 2021; Wong et al., 2013; Alahmari, 2021). The use of tight junctions is ideal when considering that the main function of the BBB is to prohibit pathogens and other toxins from entering the brain, but it becomes a challenge, when those same tight junctions exclude therapeutics from entering the brain. The adherens junctions serve to anchor the endothelial cells to each other for structural integrity (Wu D. et al., 2023; Alahmari, 2021). In Alzheimer’s disease and other neurodegenerative diseases it has been noted that the tight junctions and adherens junctions become disrupted as the disease progresses, potentially contributing to the progression of the disease, as the brain loses the protection provided by the BBB (Teleanu et al., 2022; Kaya and Ahishali, 2021). Similar observations on a compromised BBB have also been observed in those with various brain cancers including glioblastoma, though the BBB is not uniformly compromised often showing a more regionalized pattern associated with the tumor locations (Digiovanni et al., 2024; Upton et al., 2022; Kim et al., 2021; Arvanitis et al., 2020; Sarkaria et al., 2018). Although this disruption of the BBB may appear as an advantage for delivering therapies into the brain, this disruption usually occurs in the later stages of disease progression, after the ideal therapeutic intervention time. For a significant portion of neurological diseases and disorders there is minimal to no disruption of the BBB, making it necessary to deliver the desired therapy past an intact BBB.

Proceeding outwards from the blood vessels the pericytes are the cells that immediately surround the endothelial cells. These cells play a central role in neurovascular function regulating cerebral blood flow and releasing signaling factors which contribute to polarizing the end feet of astrocytes (Kaya and Ahishali, 2021; Armulik et al., 2010). The number of pericytes influences the number of tight junctions between endothelial cells impacting the BBB structure and integrity (Kaya and Ahishali, 2021; Brown et al., 2019). Defects in pericyte specific proteins can result in a compromised BBB, although this is not common to all neurological disorders or diseases (Brown et al., 2019; Sweeney et al., 2016). Although important for the structural integrity of the BBB the pericytes are not considered a directly targeted cell type for BBB entrance.

Astrocytes surround the endothelial cells in the BBB with their end feet encircling the pericytes. (Figure 1) (Alahmari, 2021). Astrocytes are the most common glial cells in the brain, infiltrating both the highly vascularized gray matter and the less well vascularized white matter. (Schiera et al., 2024). Astrocytes play a pivotal role in brain function helping with waste removal, vesicular blood flow, maintaining homeostasis regulation, nutrient maintenance and immune response (Schiera et al., 2024; Manu et al., 2023). Astrocytes further act as glucose intermediaries for neuronal cells, by absorbing circulating glucose from the blood vessels and converting it into pyruvate, which they then secrete into the extracellular spaces between the neurons, which then use this secreted pyruvate as an energy source (Beard et al., 2021; Pellerin, 2018). Astrocytes play additional roles in maintaining the BBB by regulating the water content via aquaporin proteins and the pH via astrocyte specific bicarbonate transporting proteins such as Slc4a4 (Ye Q. et al., 2024). There are various neurological disorders in which the BBB is compromised due to mutations in astrocyte specific proteins, further supporting the crucial role that astrocytes play in BBB structure and maintenance (Beard et al., 2021). Astrocytes also play a major role in neuroinflammation in the brain, by regulating the immune cells in the brain. In some diseases neuroinflammation is a symptom, which compromises the integrity of the BBB, potentially making it easier to deliver therapies across the BBB. Being able to target both astrocytes and neurons could be extremely beneficial in many neurological disorders, as it may help relieve the neuroinflammation symptoms associated with the disease, fostering a more favorable environment for the neurons.

Neurons are often considered the “holy grail” cells for therapeutic interventions, but to reach these cells therapies must find a way through or around the BBB. Neurons do not directly connect to the blood vessels, instead interacting with endothelial cells and astrocytes to form the neurovascular system. A therapy must move through endothelial cells or through the intracellular spaces before reaching the neurons. Depending on the disease or disorder targeting specific neuronal cell populations could be ideal, instead of broadly impacting all neuronal cells. This strategy is just beginning to advance with targeted viral vectors and LNPs (Hunker et al., 2025; Wang et al., 2025). For many neurological diseases and disorders a wide range of neurological cells are involved in the disease and it becomes not only necessary to target the neurons, but also the support cells which help keep the cellular environment optimal for neuronal functioning. Earlier therapies for neurological disorders focused mostly on the direct injections into the ventricles intracerebroventricular (ICV) or the cisterna magna by intracisterna magna (ICM) in order to avoid having to cross the BBB (Ye D. et al., 2024; Fischell and Fishman, 2021). Aside from the invasiveness of this process there were several major limitations of this method, which will be discussed throughout this review. The limitations of earlier delivery strategies are now being addressed and many of the improvements are having an immediate impact on the fields’ ability to meaningfully treat neurological disease and disorders.

## Navigating the labyrinth: progression of molecules through the BBB

There are several pathways that molecules and small molecule therapeutics with different physical properties can take when moving through the blood brain barrier. Several characteristics help determine the most likely pathway to be taken by a potential therapy through the BBB, including size, charge, polarity and receptor recognition sequences (Alahmari, 2021; Lochhead et al., 2020). The most direct but limited way for molecules to pass through the BBB is by passive diffusion. To utilize passive diffusion the molecule must be lipid soluble, small and uncharged (Banks et al., 2024). Many small molecule drugs (i.e.,: phenobarbital, temozolomide, and sertraline) used for treating different neurological disorders rely on this pathway, however for many therapies this pathway is not an option (Sun et al., 2023; Chowdhury et al., 2021; He et al., 2018). The use of the paracellular pathway is also limited due to the tight junctions between endothelial cells which surround the blood vessels (Kadry et al., 2020). Small molecule therapeutics are an important class of therapeutics for treating many neurological disorder, still they are not sufficient for addressing many disorders due to various limitations including specificity, potency and bioavailability. To address this unmet need biological molecules including oligonucleotides, antibodies, adeno associated virus (AAV) mediated gene therapy and others have emerged as potential therapeutics, but their transport across the BBB is more complex and challenging. Even after crossing the BBB these biological therapies must localize to the appropriate cellular type to be effective in treating the chosen neurological disorder.

For the therapies not able to use passive diffusion or the paracellular pathway the two other major pathways used are, carrier transport and receptor mediated transcytosis (Pawar et al., 2022; Papademetriou and Porter, 2015; Jones and Shusta, 2007; Burns et al., 1975). Carrier transport involves the use of a specific carrier protein that is embedded in the cells membrane and will transport specific molecules across the cells membrane. This is how glucose and amino acids are transported across the BBB (Zaragoza, 2020; Patching, 2017). However, glucose and amino acids are orders of magnitude smaller than the majority of therapies designed for neurological disease. Receptor mediated transcytosis facilitates crossing of the cell membrane for much larger sized therapies including viral vectors, some lipid nanoparticle (LNP) and delivery vehicles specifically targeting a receptor for cellular internalization (Ding et al., 2025). When designing the delivery vehicle often a specific cell receptor is targeted as the internalization receptor. The choosing of the receptor can either be an intentional choice by the scientist, for example adding a cell penetrating peptide to a LNP or protein, or it can be part of the delivery vehicles inherent characteristics, as is the case with various AAV serotypes. Engagement of the receptor by the delivery vehicle initiates transcytosis with the formation of an endosome, the endosome encapsulates the delivery vehicle and is then internalized in into the cells cytoplasm (Ding et al., 2025; Pulgar, 2018). The endosome must then transverse the cell to then fuse again with the cell membrane and be released into the brain parenchyma where it can then proceed to target the correct neuronal cell type for the designed therapy. Once in the neuronal cell the delivery vehicle and the desired therapeutic must escape the endosome for the therapy to work. If the delivery vehicle is unable to escape the endosome then it will travel to the cells lysosome where the endosome will be dissolved and the delivery vehicle and intended therapeutic will be degraded (Dowdy, 2023). In specific cases, the lysosomal localization of endosomes has been effectively implemented for neurodegenerative disorders caused by lysosomal dysfunction (Donald et al., 2025; Herman et al., 2024). For the majority of CNS therapies endosomal escape must be designed into the therapeutic, and is unique for each delivery vehicle (Dhungel et al., 2021; Lagache et al., 2012). The next several sections will focus on current advantages, challenges and clinical progress each delivery vehicle has made for transporting different therapies past the BBB.

## Trojan horses and skeleton keys: delivery strategies for getting past the BBB

### Adeno associated virus

The most well characterized and studied modality for brain delivery are viral vectors, specifically the use of adeno associated virus (AAV). AAVs are small enough to move through the blood vessels of the BBB vasculature, though until recently AAVs did not have the tropism that would allow for an intravenous injection and passage through the BBB and into the surrounding neuronal and glial cells. Traditionally to bypass the BBB, AAV has been directly injected into the cerebral spinal fluid, or by ICV or ICM injection. Direct injection methods have shown the AAV remains fairly localized at the injection site, rarely attaining the desired coverage and depth needed for many neurological disorder treatments (Ye D. et al., 2024; Hunter et al., 2025; Liu et al., 2021; Kim et al., 2014; Dirren et al., 2014). For decades the most efficient AAV for CNS delivery was AAV9, which showed levels of neuronal transduction that proved therapeutic in murine models and has been used in several human therapies (Wang JH. et al., 2024; Liu et al., 2024; Issa et al., 2023). It was initially unclear how AAV9 was able to cross the BBB, while other serotypes of AAV could not, but studies involving primary human brain endothelial cells showed that AAV9 used active transcytosis, making this transport energy dependent (Weber-Adrian et al., 2017; Merkel et al., 2017). In contrast AAV2 showed minimal ability to cross through the endothelial cells, instead remaining trapped inside vesicles, unable to leave the endothelial cells (Weber-Adrian et al., 2017; Merkel et al., 2017). The use of AAV9 has been essential in the understanding and development of different potential therapies for neurological disorders, but unmodified AAV9 has proven limited in human therapeutic applications (Wang JH. et al., 2024; Liu et al., 2024; Dayton et al., 2012). One of the reasons for the therapeutic limitations is the limited transduction levels of unmodified AAV9, which although impressive for a mouse have a much more limited effect in a human brain, which is approximately 600X the size of a mouse brain by weight (Herculano-Houzel et al., 2006). The transduction limitation is being addressed by revolutionary advancements being made in AAV capsid design through directed evolution. Some of the newer AAV capsids have been engineered to diffuse beyond the localized injection region, including the capsid used for UniQure’s impressive results in their Phase I Huntington disease trial, which when injected in the striatum is designed to spread to the cortex (Kaiser, 2025). Several of the newer AAV capsids have been designed to facilitate allow IV delivery while others have been fine-tuned for specific neuronal cell types of brain regions (Ye D. et al., 2024; Liu et al., 2021; Luo et al., 2025; Huang et al., 2024; Corti et al., 2023). The use of IV delivery will potentially allow for widespread brain delivery with a minimally invasive method, due to the AAV traveling through the entire BBB vasculature (Ye D. et al., 2024; Lee et al., 2025). Once in the vasculature of the BBB the AAV is able to enter the endothelial cells through receptor mediated transcytosis. The AAV is then encased in a vesicle that traffics to the Golgi apparatus where it is then released into the rest of the brain and can enter neuronal cells through receptor mediated endocytosis (Wong et al., 2013; Merkel et al., 2017; Moghimi and Howard, 2018). Preclinical testing has indicated that leveraging the BBB vasculature can dramatically increase the distribution of AAV in the brain, an important consideration for many neurological therapies (Ye D. et al., 2024; Lee et al., 2025).

Although AAV represents one of the most promising delivery vehicles in the brain, but there are still several challenges to be overcome to best optimize its use. Among the most complex challenges are packaging capacity, immune response to AAV, and liver targeting, these challenges represent major limitations in optimizing AAV as a delivery vehicle in patients (Ye D. et al., 2024; Corti et al., 2023). Packaging capacity is the least malleable of the challenges as AAV has a fixed capacity of about 4.7 kb of DNA, limiting the size of the desired construct (Grieger and Samulski, 2005). For many genes 4.7 kB is an adequate size, but for larger genes, like DMD or HEXA this presents a challenge (Bez Batti Angulski et al., 2023; Mahuran, 1999). Even for the gene editing CRISPR system this presents a challenge when trying to package the Cas9 and gRNA together. Strategies taken to address the packaging size have included “mini genes” which are a smaller but still partially functional version of the gene, as piloted for Rett syndrome and Duchene Muscular Dystrophy (Butterfield et al., 2025; Sadhu et al., 2023). There are also “split genes” where the gene or the editing system is split into two AAV constructs and upon entrance of both AAV constructs into the cell the pieces rejoin and are functional (She et al., 2023; Nitzahn et al., 2020). To address the immune challenge researchers are taking a dual approach, maximizing single injection coverage and designing AAVs that will trigger minimal to no immune response potentially allowing for multiple injections (Yang et al., 2022; Tse et al., 2015). The potential need for multiple injections in the CNS has been highlighted by work done in spinal muscular atrophy, in which the one-time Zolgensma injection does not completely ameliorate symptoms in some patients, and an additional treatment is needed, in this case an antisense oligonucleotide (ASO), Spinraza (Ponomarev et al., 2023; Mirea et al., 2021). The ability for multiple injections could allow for increased coverage of the brain, if needed as the patient matures. To maximize coverage from a single injection of AAV researchers have been working to de-target the liver, increasing the amount of AAV that is able to reach the brain, and optimizing AAV capsids that allow for IV injection, which enables the AAV to travel through then entire brain vasculature. These strategic improvements to AAV should create a delivery vehicle that is highly efficient at crossing the BBB and delivering the chosen therapeutic throughout the brain.

One of the most significant studies indicating that an IV injection could be used for highly efficient delivery of AAV to the brain occurred in 2018, with the creation of the capsids PHPB and its successor PHPeB. The studies were significant in that they not only created CNS specific AAVs, which efficiently crossed the BBB, they also established a methodology that has been widely adopted by others to create a variety of BBB crossing AAVs (Chan et al., 2017). The PHPeB strain was delivered by a single IV injection in a mouse and showed the most comprehensive whole brain delivery to date with minimal off targeting in other organs (Chan et al., 2017). Unfortunately, PHPeB was not translatable to primates, due to targeting a mouse specific receptor sequence, however the methodology of selective mutagenesis to create novel, neurotrophic AAVs was a major advancement. Similar strategies have been leveraged by both academia and industry to enhance neuronal tropism, decrease immune response and target cross species receptors (Mathiesen et al., 2020; Rittiner et al., 2020; Hordeaux et al., 2018). Studies have recently shown that AAV can be honed to such an exquisite specificity as to target a specific brain regions or cell type (Hunker et al., 2025; Hunker et al., 2025). This targeting specificity encompasses cell types of the brain vasculature including the smooth muscle cells and pericytes, broadening the cell targeting ability of AAVs (Ramirez et al., 2023). The increased targeting ability has been due to iterative selection rounds building upon the earlier work done to create PHPeB (Ramirez et al., 2023). These advances have been made possible due to the major initiatives in the last decade that focused on honing AAV into a therapeutically relevant delivery mechanism for CNS disorders. This focus has been in part due to the previous clinical trials, which have used AAV to limited success, in part due to AAVs limited ability to transduce neurons in an adult patient, averaging under 10% (Ye D. et al., 2024). The next-generation of AAVs are on the precipice, with the first AAV having been IV administered for Canavan disease in 2023. The Canavan trail was very limited as an n = 1 trial, and the AAV was simultaneously delivered IV and ICV, but it did show that the IV delivered AAV did not cause an acute immune response and there were some positive changes in brain morphology and behavioral milestones. However due to the simultaneous IV and ICV delivery it was difficult to ascertain to tell how effective an IV alone delivery would be. One of the major disadvantages of IV delivered AAVs is that they require a high dose to be administered due the moving through the entire circulatory system, and potential degradation issues. This higher dosage does come with potential increased safety and immune response risks. This increased risk from a high dosage will likely be re-assessed due to the recent patient death in the Capsida STXBP1 trial which used an IV delivered AAV (Corti et al., 2023; Chuapoco et al., 2023; Taylor, 2025). There is hope that with additional adjustments and dosage optimization that this new generation of AAVs will represent a substantial step forward in therapeutic delivery, and cellular specificity based on the encouraging preclinical non-human primate (NHP) studies (Chuapoco et al., 2023; Goertsen et al., 2022). For many neurological disorders an optimized AAV will allow for a significantly meaningful treatment of the disorder, while reaching specific cellular populations will both increase efficacy and reduce potential adverse effects.

### Hematopoietic stem cells

Perhaps no other potential therapy so accurately mimics the Trojan horse delivery method as that of hematopoietic stem cells (HSCs). These cells are found in an individual’s bone marrow and are the blood stem cells from which differentiate into various types of blood cells including red blood cells, macrophages, monocytes, T-cells and others (Cheng et al., 2020). Researchers have been studying the use of HSCs for decades to treat various blood and autoimmune disorders (Mancardi et al., 2024; Chen et al., 2024; Alexander et al., 2021; Ben Nasr et al., 2016). Recent developments have leveraged the BBB crossing ability of HSC derived monocytes to treat neurological disorders. In normal circumstances, monocytes do not usually enter the brain parenchyma, but in various CNS injuries or diseases where neuroinflammation is present, monocytes are able to transverse the BBB through compromised tight junctions (Biffi, 2024; Eichler and Kuehl, 2024; Ren et al., 2024). For use in treating neurological disorders autologous HSCs are genetically modified so that the monocytes and eventually the differentiated microglia-like cells will express the therapeutic proteins, which will be secreted into the brain parenchyma for neuronal cell uptake (Eichler and Kuehl, 2024; Adhikari et al., 2021). Often the desired modification is done by adding a gene of interest to the HSCs via a lentiviral vector (Eichler and Kuehl, 2024; Adhikari et al., 2021; Fumagalli et al., 2022). In HSC transplant patients there is a window following transplantation during which the monocytes are able to cross the BBB through a complex process involving initial adhesion to the endothelial cells, regulated by the CCR5 receptor (Ren et al., 2024). The monocytes then transmigrate across the endothelial cells, primarily in the post capillary venules, and enter the brain parenchyma (Mancardi et al., 2024; Ren et al., 2024; Zhang et al., 2025; Munoz-Castro et al., 2023). After entering the brain parenchyma the monocytes differentiate into macrophages and microglia-like cells (Reu et al., 2017). These microglia-like cells are then able to secrete therapeutic proteins into the surrounding intracellular fluid from which neuronal cells can import these therapeutic proteins (Chen et al., 2024; Rahimi et al., 2024). HSC technology has proven extremely promising in the last 5 years with four different disease modify therapies approved, and several more in the clinical and preclinical pipeline.

The strategy for gene edited HSCs has been successfully used in several neurological and non-neurological diseases. Since 2022 the FDA has approved several modified HSC treatments, two of these treatments were for CNS lysosomal storage disorders, Skysona for cerebral adrenoleukodystrophy (CALD) and Lenmeldy for metachromatic leukodystrophy (Philippidis, 2024a; Keam, 2021). The other approvals were for Casgavy, for sickle cell disease and Zynteglo for beta-thalassemia (Eichler and Kuehl, 2024; Fumagalli et al., 2022; Fumagalli et al., 2025; Singh et al., 2024). These approvals represent the first of many treatments that are in development for HSCs. Patients that have been treated with HSCs have shown remarkable recovery and survival rates indicating the extreme promise of this delivery method (Fumagalli et al., 2025; Biffi et al., 2013). One of the major benefits of HSC therapy is the possibility to be a “one and done” treatment, as the modified HSCs have shown remarkable durability during the continued follow-up visits for the treated patients (Singh et al., 2024; Hardouin et al., 2025; Leonard and Tisdale, 2024; Raymond et al., 2019; Barciszewski and Legocki, 2001). Several other neurological disorders are embracing the HSC technology with promising preclinical studies, including Angelman syndrome, Rett syndrome, and SYNGAP1 syndrome amongst others (Adhikari et al., 2021; Rahimi et al., 2024; Anderson et al., 2024; Beegle et al., 2020). There continues to be risks to this approach, among them are unintended genetic modifications, either by lentiviral integration in the vicinity of proto-oncogenes, off-target gene editing if CRISPR is used, and a potentially poor reaction to the preconditioning regimen, which makes space for the newly implanted HSCs (Ikeda et al., 2018; Zulu et al., 2018). For Skysona there were some patients that developed myelodysplastic syndrome (MDS) and acute myeloid leukemia (AML), which triggered a warning from the FDA marking the treatment as carrying a risk of cancer development (Philippidis, 2024b). This particular concern has been addressed by changing the specific gene promoter used in Skysona, MNDU3, to a different promoter in future treatments (Puig-Serra et al., 2025). It should be noted that MDS risk was not generally related to HSC therapy as other HSC therapies using different promoters have not observed this level of MDS occurrence (Puig-Serra et al., 2025). Additional unknowns with HSC therapy are how long the edited microglia-like cells last once in the brain, with current studies indicating many years, and if later monocyte migration to the brain is possible to replenish the initial microglia-like cells, or if this replenishment is even necessary (Zhang et al., 2025; Reu et al., 2017; Yoo and Kwon, 2021). For many patients the symptoms of the disease are so severe that the treatment benefits outweigh the associated risks. As HSC therapy continues to be refined it is expected that various modifications and improvements will decrease the associated risks opening the pathway for even more HSC related treatments.

### Cell penetrating peptides (CPPs)

For delivery modalities the cell penetrating peptide (CPP) could be considered the skeleton key of delivery approaches, highly versatile they have the ability to be used by themselves or added to the other delivery modalities. CPPs are short sequences of amino acids, usually under 20, that have been derived from a variety of sources, including viruses, endogenous proteins, artificially designed (Blades et al., 2023; Xie et al., 2020). There are over 1800 different cell-penetrating peptides that have been verified to carry different therapeutic cargos into cells, including small molecules, nucleic acids and proteins (Ghorai et al., 2023; Bottens and Yamada, 2022). Of these 1800 only a selected few have been shown to enter neuronal cells and even fewer have been able to cross the BBB (Blades et al., 2023; Bottens and Yamada, 2022). CPPs are able to cross the BBB through a few major methods, adsorptive mediated transcytosis, receptor mediated transcytosis and direct penetration (Blades et al., 2023; Xie et al., 2020; Zou et al., 2013; Herve et al., 2008). The methods of absorptive mediated transcytosis and direct penetration could be considered “non-specific” in that it is not a specific set of amino acids that drives the passage through the cell membrane, but instead a specific set of characteristics inherent to that CPP, which can be found in a variety of CPPs with differing amino acid sequences. The method involving receptor mediated transcytosis instead relies on a very specific set of amino acids that is recognized by a specific cell membrane receptor, and this method will be covered in the next section (Barker et al., 2024; Xiao and Gan, 2013; Wiley et al., 2013).

In adsorptive mediated transcytosis the passage of the CPP-cargo is dependent upon the CPPs positive charge, which interacts with the negatively charged components on the cell membrane. The CPP-cargo is transported into the cell by a clathrin or caveolar vesicle, this vesicle then transports the CPP-cargo through the endothelial cell where it is released into the brain parenchyma and taken up by neuronal or glial cells. An excellent example of this type of CPP are the poly-arginine based CPPs, which have been shown to transport nucleic acids across the BBB (Holm et al., 2022; Kumar et al., 2007). For the peptides that rely more on lipid solubility several models exist for how they allow the uptake of the CPP-cargo. One model involves the formation of inverted micelles, where the CPPs create small hydrophilic pockets that help shuttle the CPP and their attached cargo across the cell membrane (Kawamoto et al., 2011). Another model involves pore formation, with two main models: the Barrel-Stave Model, where CPPs form helical structures lining the pores inside with hydrophilic regions and interact with the membrane via hydrophobic parts; and the Toroidal Model, where CPPs bend the membrane lipids themselves, combining lipids and peptide to create stable pores (Shin et al., 2014). Examples of lipid soluble CPPs include TAT, MAP and TP10. For many CPPs the exact method of transport across the cell membrane and the BBB remains an area of active research, especially for CPPs that are designed in the laboratory and not derived from a natural source.

One of the challenges with using CPPs when designing a potential neurological therapy is their inconsistency. A single CPP is not able to transport every cargo into cells that it is attached to, and often a CPP that works in mice will not translate to humans. The CPP, TAT, is one such example, it has shown the ability to cross the BBB with an attached cargo in mice, but this has been inconsistent and cargo dependent in humans (Zou et al., 2013; Wu MC. et al., 2023; Bailus et al., 2016). One explanation for the inherent inconsistency in CPPs could be due to how the cargo affects their ability to interact with the endothelia cell membrane. For example when a CPP is attached to a protein that protein will fold a certain way, impacting the CPPs ability to interact with the endothelial cell membrane, and each protein will fold slightly differently impacting the CPPs efficacy. One way in which this inconsistency is being addressed is to use the CPP as a complimentary additive to either AAVs or LNPs (Vargas et al., 2024; Pardridge, 2023). Multiple preclinical studies have shown that BBB penetrance and distribution of AAVs and LNPs can be increased when a CPP is added (Shi et al., 2024; Teixeira et al., 2023; Meng et al., 2021). A major benefit of this strategy is that the inconsistency of the CPP is reduced. When attached to an AAV or LNP, the CPP will have a fixed orientation, making its presentation to the endothelial cell membrane cargo independent, allowing for more reproducible cell membrane or receptor mediated interactions. Even with these challenges there have been several successful uses of CPPs crossing the BBB in preclinical models, and in clinical trials (Xie et al., 2020).

Several CPPs that rely on Adsorptive-mediated transcytosis (AMT) for navigating through the BBB have shown promise both in preclinical studies and in clinical trials. TAT, derived from HIV is arguably the most well-known from this class of CPPs. TAT was used in the ESCAPE-NA-1 Phase 3 clinical trial to deliver an oligopeptide into the brain of those undergoing aneurysm repair surgery in the hope of reducing ischemic strokes that can occur during surgery (Goyal et al., 2025; Zhou, 2021). Although the treatment itself did not work as intended the study did show that the TAT peptide was able to cross the BBB in these human patients. Another promising CPP, Penetratin, derived from Antennapedia a protein found to play a role in insect development (Dupont et al., 2011). Penetratin has shown promise in transporting cargos across the BBB in preclinical studies, and has also been added to LNPs to enhance their BBB crossing ability (Blades et al., 2023; Chen et al., 2012). It is expected that the variety, efficiency and predictability of CPPs will expand dramatically with the advancements in artificial intelligence. The use of Alpha-fold to predict CPP-Cargo folding could dramatically improve predictions on which CPP to add to a specific cargo, enhancing the predictability of how the CPP would be presented to the cell membrane once attached to a chosen protein (Jumper et al., 2021). The synergy of artificial intelligence and massive parallel screening may help CPPs fulfill their early promise for direct therapeutic delivery and advance their integration into other delivery vehicles.

### Receptor mediated targeting

Receptor Mediated Targeting (RMT) leverages the different receptors on the endothelial cells membrane for crossing the BBB by receptor mediated transcytosis (Pawar et al., 2022; Xiao and Gan, 2013). In many ways RMT is similar to the use of CPPs except the design process involves designing a ligand that is recognized by a specific receptor, instead of selecting a sequence of peptides based on their chemical or physical properties, or by conducting a random screen to generate new ligands. Once the ligand fused therapeutic is taken into the endothelial cell by the desired receptor it enters an endosome which for an effective therapy must then fuse with the abluminal membrane and release its contents into the brain parenchyma. Several different ligands have been tested in human clinical trials for therapeutic delivery to the brain.

One of the most widely targeted receptors for crossing the BBB is transferrin. Over a decade of research has supported targeting the transferrin receptor to deliver therapeutics from proteins to nucleic acids to nano-particles. This work has shown promise in preclinical studies for Alzheimer’s disease with the transferrin receptor targeting ligand being attached to antibodies, erythropoietin and other potential therapeutics (Jagadeesan et al., 2024; Yang et al., 2023; Sumbria et al., 2013; Boado et al., 2010). The transferrin receptor is highly expressed on the surface of the BBB endothelial cells. Several strategies have been used in which the transferrin protein, transferrin receptor antibodies, or peptides that bind to the transferrin receptor have been added to the therapeutic or the delivery vehicle (Huang et al., 2024; Wiley et al., 2013; Sumbria et al., 2013; Boado et al., 2010). Similar to what was previously discussed in the CPP section transferrin receptor targeting ligands can be added to other delivery vehicles to increase their ability to cross the BBB. One unique method from Denali Therapeutics added the transferrin targeting ligand to the Fc domain of an antibody and then conjugated an ASO to this antibody, greatly enhancing the ability of the ASO to enter and distribute in the brain of a NHP (Barker et al., 2024; Khoury et al., 2025). One of the challenges using this technology is that transferrin is expressed on other cell surfaces beyond the brain endothelial cells, creating the potential for off-target delivery, the effects of which would need to be evaluated for each treatment. The risk of off-target delivery is a common risk for many delivery methodologies, as very few receptors are completely unique to a specific organ or cell type. Another challenge is that not all therapeutic cargos will be amenable to conjugation with a transferrin targeting delivery vehicle or function properly after conjugation. Even with these challenges these ligands have advanced into the clinic and are yielding promising early results.

There are several clinical trials are underway using receptor mediated transcytosis as the brain delivery strategy by targeting different receptors that are enriched in the BBB. Targeting the transferrin receptor has shown promise in several clinical trials, one of which has been approved for patient use in Japan. This therapy, IZCARGO, is made by JCR pharmaceuticals was approved for use treatment of Mucopolysaccharidosis type II, in 2021 (Okuyama et al., 2021; Okuyama et al., 2019). IZCARGO is an enzyme replacement therapy in which, iduronate-2-sulfatase, is attached to a transferrin receptor targeting antibody and administered via an IV infusion. JCR Pharmaceuticals, has several additional therapies using this technology in their clinical pipeline. Denali Therapeutics is another company with several transferrin based therapies currently in clinical trials, the most advanced of those being for Hunter Syndrome (Arguello et al., 2021). Another receptor that has been targeted with clinical success is the low-density lipoprotein receptor-related protein 1 receptor (LRP-1) (Xiao and Gan, 2013). The LRP-1 receptors are abundant on BBB endothelial cells, making this an ideal target for engagement and receptor mediated transcytosis across the BBB (Nikolakopoulo et al., 2021; Sagare et al., 2012). Angiopep-2 a peptide that specifically targets LRP-1 has been used in several clinical trials for glioma with some success, and has a positive safety profile (Xiao and Gan, 2013; Zhu et al., 2021; Kurzrock et al., 2012). The success of these current clinical trials and the flexibility of receptor mediated targeting ligands makes this strategy a potential future leader in BBB delivery for neurological diseases and disorders.

### Lipid-nanoparticles

Lipid nanoparticles (LNPs) have recently become a promising brain delivery vehicle advancing beyond their initial use in the liver. An LNP is a small 50–200 nm sphere composed of pegylated lipids and other molecules including peptides and cholesterols which encase the desired therapeutic (Cullis and Felgner, 2024). LNPs primary mode of entrance into a cell is by endocytosis, which necessitates the endosomal escape of the cargo, often facilitated by ionizable lipids that are part of the coating of the LNP (Han et al., 2021; Swingle et al., 2021). The use of LNPs as a delivery vehicle works extremely well when the cargo is targeted for the cells cytoplasm, and for mRNA and pure proteins this is often the case. Although LNPs received FDA approval over 30 years ago for delivery of chemotherapeutic agents in was not until 2020 that LNPs became high profile with the approval of Pfizer and Moderna’s COVID-19 vaccines, both of which utilized LNPs to encapsulate their mRNA vaccines (Zhang et al., 2023; Hou et al., 2021). The total number of vaccines given for Pfizer and Moderna exceeded 2 billion, and LNPs were also used for the Pfizer and Moderna COVID-19 booster shots, indicating that LNPs have an excellent safety profile for single and multiple dose administrations (Firouzabadi et al., 2023). Outside the COVID-19 vaccines there are several ongoing clinical trials using LNPs for delivery to blood cells, lungs, liver, with over eighty trials listed on clinicaltrials.gov. LNPs offer several advantages over other delivery systems, less stringent size restrictions, less integration risk into the hosts DNA, and the ability for reduced immune response risk (Wang J. et al., 2024; Mehta et al., 2023; Lee et al., 2023). It is expected that the use of LNPs will continue to expand as they are optimized for additional organ and tissue deliveries.

Initially LNPs were targeted to the liver, but rapid progress has been made in the preclinical space for adapting LNPs for use in brain delivery. The physicochemical properties of LNPs, including size, surface charge, and targeting ligands, can be tailored to improve their uptake by brain endothelial cells (Yuan et al., 2024; Khare et al., 2023; Jose et al., 2014). Once at the BBB, LNPs can interact with membrane-bound receptors or exploit cellular uptake pathways of clathrin- or caveolin-mediated endocytosis to enter the CNS (Hersh et al., 2022). Additional studies have indicated that LNPs can transiently disrupt tight junctions, facilitating paracellular transport without causing lasting damage to BBB integrity (Zha et al., 2024). Several studies in mice targeting glioblastoma have shown LNPs to be able to cross the BBB and specifically target tumors (Kaur et al., 2024; Lai et al., 2024; Herrera-Barrera et al., 2023). The LNPs in these and other studies were modified to display specific ligands including RVG29, T7, AP2, and mAPOE, on their surface to enable BBB crossing from an IV injection (Kaur et al., 2024; Han et al., 2025). More recent advances have focused on generating a library of LNPs that are able to cross the BBB, with one study creating 72 different “strains” of LNPs that could cross the BBB (Wang et al., 2025). These new LNPs dubbed blood-brain-barrier-crossing lipid nanoparticles (BLNPs) were designed to use receptor mediated transcytosis, absorptive-mediated transcytosis and carrier mediated transcytosis depending on which ligands were displayed on the LNP surface. This study further helped elucidate the role that caveolae and γ-secretase play in the transcytosis process, allowing for the LNP to transverse the endothelial cell toward eventual entrance into the glial and neuronal cells. Perhaps most significantly from a therapeutic viewpoint this study examined the safety profile of administering multiple doses of the BLNPs, with no obvious toxicity observed after multiple injections (Wang et al., 2025). The ability to administer multiple injections of LNPs would dramatically alter the neurological therapeutic landscape potentially resulting in more comprehensive brain coverage from the initial set of IV injections, while also facilitating later injections as needed. For neurodevelopmental disorders where one aims to treat as early as possible, the ability to administer additional treatments as the child grows and develops could be crucial to sustained efficacy of the treatment. For adult patients multiple LNP administrations could also prove beneficial as new studies are showing the brain continues to produce neuronal stem cells throughout a person’s lifetime, resulting in a reservoir of cells that might need to be treated after an initial treatment (Dumitru et al., 2025). Though still in the preclinical stage LNPs are moving rapidly toward clinical application for neurological disorders, positioning them to be part of the next wave of BBB crossing delivery vehicles.

### Through the main gate: physical bypass of the BBB

A physical approach that can complement many of the previously described delivery strategies is focused ultrasound (FUS). Focused ultrasound creates a temporary physical change in the BBB through the use of targeted ultrasound and microbubbles (Papademetriou and Porter, 2015). Focus ultrasound involves the use of IV injected microbubbles which oscillate when targeted by the focused ultrasound, putting pressure on the BBB blood vessel walls causing the tight junctions between the endothelial cells to loosen, allowing for a temporary entrance of therapeutics that would not normally occur (Papademetriou and Porter, 2015). This methodology mimics what is often found in late stage neurodegenerative diseases where the BBB is weakened and the tight junctions are loosened, the difference being that focused ultrasound causes a temporary weakening where in neurodegenerative disorder that weakening is a persistent symptom of the disease (Roberts et al., 2022; Sweeney et al., 2018). For therapies that would require a single dose, including viral vectors or gene engineering proteins the focused ultrasound can be used to enhance delivery allowing for greater distribution and deeper penetration into the brain. Focused ultrasound might be less useful for treatments that require repeated doses including ASOs, ERTs, and small molecules as regular weakening of the tight junctions could eventually compromise the integrity of the BBB (Katz et al., 2023). There are several clinical trials in progress that use focused ultrasound including those for glioblastoma and Alzheimer’s disease. These trials show early promise and the fact that focused ultrasound can be leveraged as a method to enhance other delivery methodologies makes it an exciting future prospect for using in more neurological therapies to enhance delivery.

### Hoist the banners: the future of BBB delivery

The advancements of gene editing and brain delivery have accelerated at an astounding pace in the last 30 years. For the first time in human history the essential elements for disease modifying therapies across a broad range of neurological disorders are available, and advancing toward the clinic. The various delivery modalities presented in this review represent and expanding field of study, with significant focus and support from academic and industry partners. One of the most exciting developments in the delivery field in the last decade are the overlaps and synergies that are being uncovered amongst the different delivery modalities. Using CPPs to compliment AAV and LNP delivery systems, incorporating FUS into the initial delivery to enhance overall brain distribution is just the beginning of what will likely become a multilayered and synergistic approach toward therapeutic delivery to the brain. The entire field is expected to undergo major advancements with the progression of artificial intelligence and the ability to design novel peptides, AAV capsids, and to predict the targeting and immune profile of these new constructs. The beginning of this new era has just been glimpsed at with the publication of the most recent wave of articles from the BRAIN Initiate focused on honing AAV into a cell specific delivery vehicle that can target very specific neuronal cell types, from astrocytes to microglia to excitatory neurons. The advancement of these delivery systems will also enhance our knowledge of basic brain development, architecture, and neurological disease progression. These advancements will help inform the continued development of different delivery modalities ensuring these modalities can accommodate the vast array of potential therapies as they emerge.
